# Cutaneous Periumbilical Fibroadenomas: A Rare Case of Ectopic Breast Tissue

**DOI:** 10.7759/cureus.17523

**Published:** 2021-08-28

**Authors:** Alexis Hilts, Reba Suri, Mac Machan, Upinder Singh

**Affiliations:** 1 Dermatology, University of Nevada, Los Vegas (UNLV) School of Medicine, Las Vegas, USA; 2 Dermatology, University of Kansas Health System, Kansas City, USA; 3 Dermatology, Southern Hills Hospital and Medical Center, Las Vegas, USA; 4 Dermatology, Vivida Dermatology, Las Vegas, USA; 5 Internal Medicine, Southern Hills Hospital and Medical Center, Las Vegas, USA

**Keywords:** ectopic breast tissue, accessory breast tissue, ectopic fibroadenoma, fibroadenoma, fibroadenoma of the axilla, supernumerary nipples

## Abstract

Ectopic breast tissue is the presence of retained breast tissue along the embryologic mammary ridge, also known as the milk line. Accessory tissue can be located anywhere along or outside the anatomic milk line extending from the axilla to the groin. Ectopic breast tissue can undergo the same physiologic and pathologic changes seen in normal breast tissue, such as fibroadenomas, fibrocystic changes, and malignancy. The wide range of clinical presentations and symptomatology can pose a significant diagnostic challenge, and clinicopathologic correlation is key in establishing the diagnosis. In this report, we review the clinical and histopathologic findings in a rare case of cutaneous periumbilical fibroadenomas in a 25-year-old female.

## Introduction

Ectopic fibroadenoma is a benign tumor of the breast that is most commonly found in the axilla but can occur in any location along the anatomic distribution of the embryologic milk line [1-3]. Lesions range from asymptomatic to severely tender, erythematous, and edematous growths [2,4]. Ectopic breast tissue is responsive to hormonal stimulation and therefore may be asymptomatic until times of physiologic (ie, puberty, menstruation, pregnancy) or pathologic fluctuations in the level of reproductive hormones [1-3]. Ultrasound or mammography can be used during the initial evaluation, but histopathologic findings consistent with the presence of glandular, nipple, and/or areolar breast tissue are required for diagnosis [4,5]. Accurate diagnosis is important as fibroadenomas have an almost two-fold increased risk for malignant transformation and therefore should undergo the same rigorous breast cancer screening as pectoral breast tissue [6,7].

In this case report, we discuss a young woman who presented with a three-year history of progressively enlarging periumbilical growths. Excision of these nodules demonstrated glandular epithelial and stromal hyperplasia confirming the diagnosis of ectopic fibroadenomas.

## Case presentation

**Figure 1:**
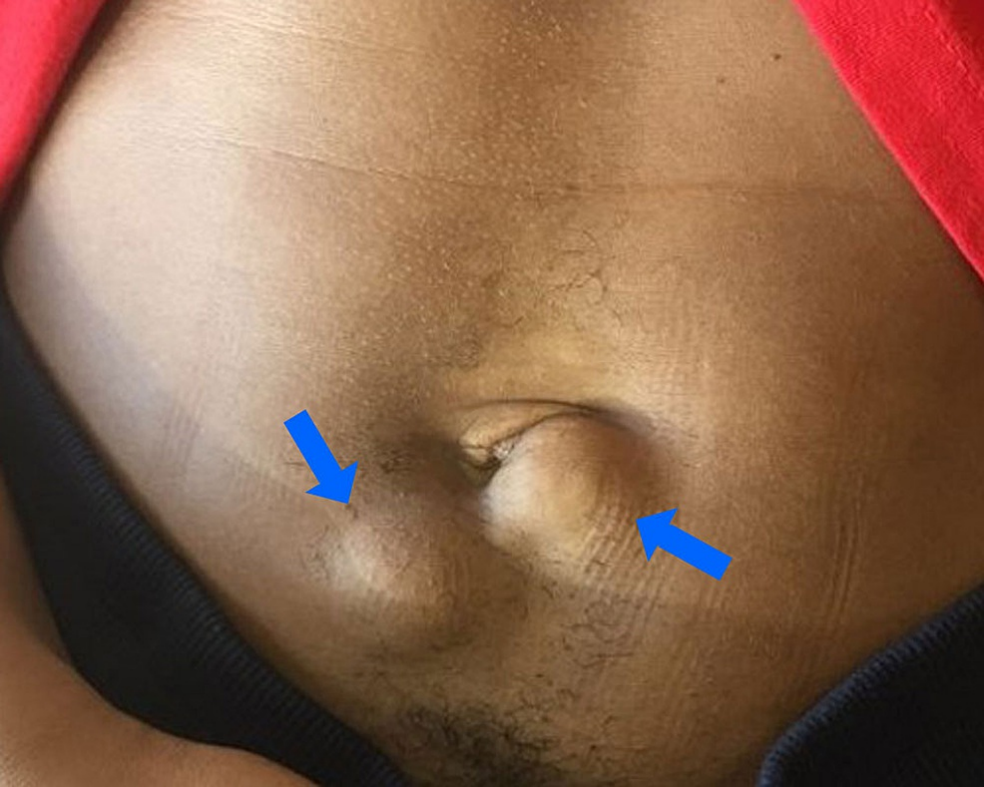
Clinical presentation of cutaneous fibroadenomas in a 25-year-old female. Two non-tender ~2 x 2.5 cm flesh-colored well-circumscribed, firm, mobile nodules located in the periumbilical region.

A 25-year-old African-American female presented with a three-year history of enlarging growths in the periumbilical region. The growths originally began as small subtle bumps under the skin and slowly enlarged over time. They were not tender to palpation but were occasionally painful with movement and during menstrual cycles. Physical examination revealed two flesh-colored, ~2.0 x 2.5 cm rubbery, non-tender, mobile, well-circumscribed and non-reducible nodules that appeared confined to the subcutaneous tissue (Figure 1). The remainder of the patient’s physical examination and review of systems was negative.

**Figure 2:**
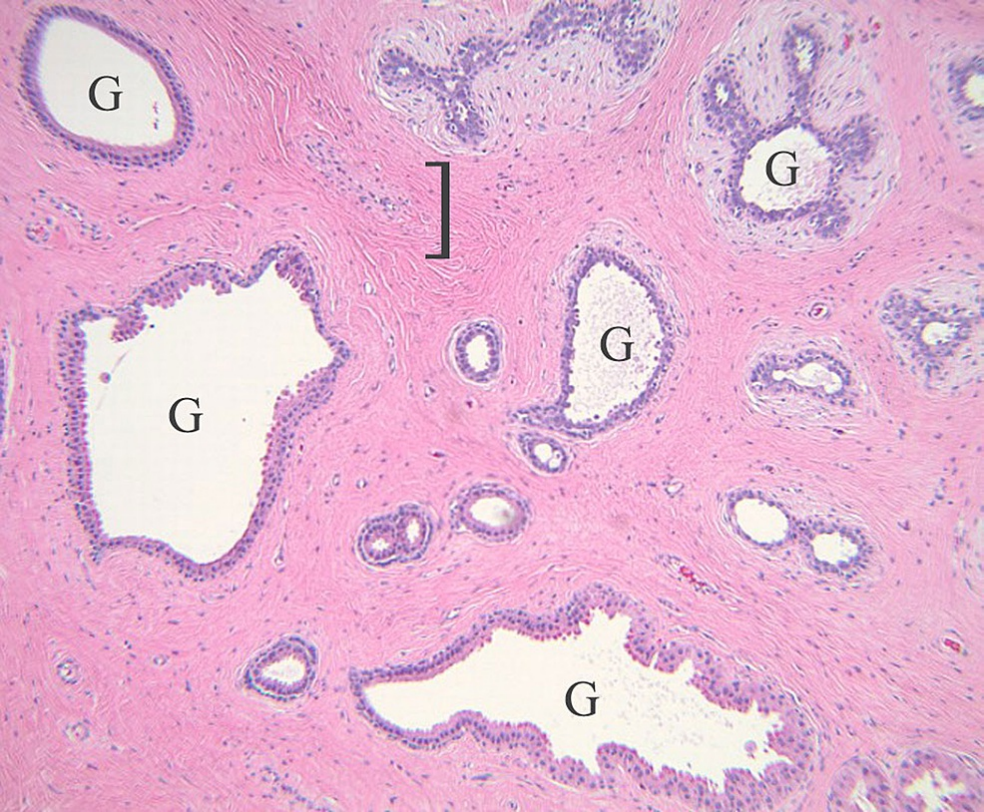
Histologic findings of a periumbilical cutaneous fibroadenoma in a 25-year-old female. Excisional biopsy demonstrating a well-circumscribed neoplasm composed of stromal fibrosis (as shown by the brackets) and glandular epithelial hyperplasia (G) in a pericanalicular to primarily intracanalicular pattern, with no evidence of stromal or epithelial dysplasia/atypia. (Hematoxylin and eosin: x100).

Excisional biopsy of a periumbilical lesion revealed a well-circumscribed neoplasm comprised of stromal fibrosis and glandular epithelial hyperplasia in a pericanalicular to primarily intracanalicular pattern, with no evidence of stromal or epithelial dysplasia/atypia (Figure 2).

The patient's histopathological and clinical findings were most consistent with a diagnosis of ectopic cutaneous fibroadenomas. 

## Discussion

During embryonic development, breast tissue and glands grow along mammary ridges [1]. Mammary ridges are termed “milk lines” and extend from the axilla to the inguinal region [2]. During normal development, mammary ridges form breasts in the pectoral region and their remainder regresses [1,2]. It is theorized that ectopic breast tissue can develop if the remaining portions of the mammary ridges fail to regress [1-3,6]. Ectopic breast tissue can occur anywhere along the mammary ridge but is most commonly found in the axilla; findings outside of the axilla are rare [1-3]. Ectopic breast tissue occurs in 0.4%-6% of the population, with the highest prevalence seen in females of Japanese descent [2,5]. Patients may not be aware of ectopic breast tissue until later in life as it responds to hormonal changes and typically develops during puberty, pregnancy, or lactation [1-3]. Periodic episodes of increased swelling, tenderness, and growth are often reported during menstruation [5]. 

Ectopic breast tissue can undergo physiologic and pathologic changes seen in normal breast tissue such as mastitis, fibrocystic changes, and fibroadenoma [1,2]. Fibroadenomas are benign tumors that occur when there is a concomitant proliferation of stromal and epithelial elements [1,8]. Depending on the proportion of these two elements, lesions can be further classified as intracanalicular and/or pericanalicular [8]. In intracanalicular fibroadenomas, stromal proliferation leads to the distortion of ductal architecture, compressing ducts into slits [1,8,9]. In pericanalicular fibroadenomas, native ductal architecture is maintained as the fibrous stroma proliferates around the ducts, leaving them round and intact [1,8]. Ectopic fibroadenomas have a variable presentation but tenderness, irritation, erythema, and milk secretion have all been reported [2]. The ectopic fibroadenomas seen in our patient displayed a relatively benign course of progressive indolent enlargement associated with intermittent pain due to movement. 

The differential diagnosis, in this case, includes fibroadenoma, lipoma, cyst, and although less likely a cutaneous metastasis from an underlying malignancy [2]. Depending on anatomic location, various imaging modalities can be useful in the evaluation of non-specific soft tissue masses [4]. The echotexture, vascularity, and contour of a lesion can be evaluated using ultrasonography [2]. Sonographic findings of fibroductal tissue and fat lobules resembling normal breast tissue can support the diagnosis of ectopic breast tissue [4]. MRI can also be performed for atypical cases, or if there is a concern for malignancy to delineate the depth of invasion [4]. Axillary lesions should be further evaluated with mammography [4]. Definitive diagnosis is achieved with fine-needle aspiration cytology or biopsy [4]. Most patients with ectopic breast tissue are asymptomatic and therefore do not require treatment if histologic findings are benign [4]. Surgical excision is the treatment of choice for lesions that are symptomatic, have histologic features concerning malignant transformation, or simply in cases where cosmetic removal is desired [4].

This report demonstrates a unique case of ectopic fibroadenoma diagnosed in a patient with multiple growths in the periumbilical region. Histopathology in addition to clinical presentation helped to establish the diagnosis of fibroadenomas in this patient. Both lesions were completely removed via excision with no evidence of recurrence to date.

## Conclusions

Ectopic fibroadenomas are rare benign tumors containing both fibrous and glandular tissues. They can occur anywhere along the milk line but are seen most commonly in the axilla. Ectopic fibroadenomas can be asymptomatic or produce a variety of hormone-sensitive symptoms such as tenderness, irritation, erythema, and milk production. Ultrasound, mammography, or MRI can be used for initial evaluation. However, characteristic histopathologic evaluation is required for definitive diagnosis. Clinicopathologic correlation is critical in establishing the diagnosis, stratifying risk for malignant transformation, and guiding treatment for this obscure disease entity.
